# Strategies to promote language inclusion at 17 CTSA hubs

**DOI:** 10.1017/cts.2024.13

**Published:** 2024-03-25

**Authors:** Linda Sprague Martinez, Cristina Araujo Brinkerhoff, Riana C. Howard, James A. Feldman, Erin Kobetz, J. Tommy White, Laurene Tumiel Berhalter, Alicia Bilheimer, Megan Hoffman, Carmen R. Isasi, Cynthia Killough, Julia Martinez, Johanna Chesley, Arshiya A. Baig, Capri Foy, Nadia Islam, Antonia Petruse, Carolina Rosales, Michele D. Kipke, Lourdes Baezconde-Garbanati, Tracy A. Battaglia, Rebecca Lobb

**Affiliations:** 1 Boston University School of Social Work, Boston, MA, USA; 2 Boston University Clinical and Translational Science Institute, Boston, MA, USA; 3 Boston Medical Center Health System, Boston, MA, USA; 4 University of Miami Clinical and Translational Science Institute, Miami, FL, USA; 5 North Carolina Translational and Clinical Sciences (NC TraCS) Institute, University of North Carolina, Chapel Hill, NC, USA; 6 Dept of Family Medicine, Jacobs School of Medicine and Biomedical Sciences, University at Buffalo, Buffalo, NY, USA; 7 University at Buffalo Clinical and Translational Science Institute, Buffalo, NY, USA; 8 University of Minnesota Clinical and Translational Science Institute, Minneapolis, MN, USA; 9 Department of Epidemiology and Population Health, Albert Einstein College of Medicine, New York, NY, USA; 10 The Harold and Muriel Block Institute for Clinical and Translational Research (ICTR), New York, NY, USA; 11 Clinical and Translational Science Center, University of New Mexico Health Sciences Center, Albuquerque, NM, USA; 12 Department of Medicine, University of Chicago Center for Institute for Clinical and Translational Science, University of Chicago, Chicago, IL, USA; 13 Wake Forrest University School of Medicine Clinical and Translational Science Institute, Winston-Salem, NC, USA; 14 New York Langone University Clinical and Translational Science Institute, New York, NY, USA; 15 Clinical and Translational Science Institute Office of Clinical Research, University of California Los Angeles, Los Angeles, CA, USA; 16 University of Florida Clinical and Translational Science Institute, Gainesville, FL, USA; 17 Southern California Clinical and Translational Science Institute (SC CTSI), Los Angeles, CA, USA

**Keywords:** Clinical and translational research, linguistic inclusion, language justice, limited english proficiency

## Abstract

The prioritization of English language in clinical research is a barrier to translational science. We explored promising practices to advance the inclusion of people who speak languages other than English in research conducted within and supported by NIH Clinical Translational Science Award (CTSA) hubs. Key informant interviews were conducted with representatives (*n* = 24) from CTSA hubs (*n* = 17). Purposive sampling was used to identify CTSA hubs focused on language inclusion. Hubs electing to participate were interviewed via Zoom. Thematic analysis was performed to analyze interview transcripts. We report on strategies employed by hubs to advance linguistic inclusion and influence institutional change that were identified. Strategies ranged from translations, development of culturally relevant materials and consultations to policies and procedural changes and workforce initiatives. An existing framework was adapted to conceptualize hub strategies. Language justice is paramount to bringing more effective treatments to all people more quickly. Inclusion will require institutional transformation and CTSA hubs are well positioned to catalyze change.

Clinical and translational research infrastructure, policies, and practices have not kept pace with demographic shifts in the United States (US) population. The number of households in which English is not the primary language has tripled since 1980 and, today, nearly 68 million (1 in 5) people speak a language other than English at home [1]. This number is significantly higher in urban areas. However, rural communities and midsized cities and towns have also seen an increase in linguistic diversity over the last two decades [1,2]. Despite linguistic changes across US cities and towns, the clinical and translational research workforce has remained largely monolingual, English, which poses a significant barrier to translational research [3,4].

The linguistic diversity of the US coupled with persistent health inequities [2,5,6] has led to calls for researchers to shift from a focus on *language access* to intentional efforts to advance language justice. All federally funded programs are required to provide access to people who have limited English proficiency (LEP) [3], defined as,

*Language assistance that results in accurate, timely, and effective communication at no cost to the LEP individual. For LEP individuals, meaningful access denotes access that is not significantly restricted, delayed or inferior as compared to programs or activities provided to English proficient individuals* [7].

In this definition, “meaningful access denotes that access is not significantly restricted, delayed or inferior.” However, the criteria for “significantly,” and who defines said criteria, is not clear and, thus, left to interpretation, including the potential for a two-tiered system wherein some potential participants face more restricted, as opposed to equitable, access to information. The shift toward *language justice* strives for equitable access and recognizes that power is inherent in language, culture, and self-expression [7].

*Language justice is rooted in a history of resistance by communities and peoples whose voices and cultures have been suppressed for generations. Language justice is an alternative to that historical pattern of disenfranchisement and oppression. It affirms the fundamental rights of individuals and communities to language, culture, self-expression, and equal participation* [8].

The prioritization of English language in clinical research is an injustice that hinders translational science [9]. Samples that are biased toward English-speaking individuals do not necessarily generalize to wider patient populations [7]. However, the lack of linguistic diversity and exclusion of non-English-speaking populations from research studies remains prevalent; and barriers to equitable research participation persist [10–14]. Solutions that lead to diverse linguistic representation among research participants are critical for identifying and increasing access to specialized novel treatments that are effective for diverse racial, ethnic, and cultural groups, and may, thus, serve to help reduce racial and ethnic inequities in health.

To inform efforts to advance language justice in clinical research, researchers from the Boston University Clinical Translational Science Institute (CTSI) received pilot funds to assess and describe promising practices to advance linguistic diversity in research conducted within and supported by NIH Clinical Translational Science Award (CTSA) hubs, which are also referred to as CTSIs. The CTSA program is specifically designed “to bring treatments to more patients quickly [15].” Moreover, the program, since its inception, has included an explicit focus on community engagement and also elevates the integration of what NIH defines as “special populations” (which includes those who speak languages other than English) [16]. As such, there may be important lessons to learn from CTSA hubs, particularly those situated in areas with linguistically diverse communities.

The research reported in this paper specifically explored strategies being implemented by CTSA hubs to advance linguistic diversity and challenges as well as CTSA hub recommendations for improving the inclusion of people who speak languages other than English in research. The research methods are presented, followed by identified strategies. In addition, recommendations from sites are highlighted.

## Methods

Key informant interviews were conducted with CTSA hubs (n = 17) in late fall and winter of 2022 by the study principal investigator (PI) (LSM) and a doctoral-level graduate student (CAB). The purpose of the interviews was to explore CTSA hub efforts to include individuals who speak languages other than English and to increase overall linguistic diversity in clinical and translational research as well as to identify lessons learned, promising practices, and recommendations for advancing inclusion. The study was reviewed by the Boston University, Charles River Campus Institutional Review Board and determined to be non-human subjects research protocol #6688X.

### Sampling and recruitment

Purposive sampling was used to identify CTSA hubs focused on language inclusion. A doctoral-level student (RH) used the National Center for Advancing Translational Sciences website to identify CTSA hubs funded in 2021 and 2020 (*n* = 63). The website of each identified CTSA hub was scanned for key phrases (“language inclusion and access,” “non-English speaking and immigrant communities,” “racial and ethnic minority populations,” “racial and ethnic disparities or inequities”) that indicated programmatic elements and services that support linguistic inclusion. The details of identified efforts were further explored on the website. Seven hubs were identified with programs and services explicitly focused on language inclusion and 17 additional hubs that referenced the engagement of special populations were also identified. As such, total of 24 hubs (38%) made up the total sample. Doctoral students (CAB & RH) identified key informants from each hub using the website. In cases where a specific contact was not listed an email was sent to the CTSI website portal to identify a key informant.

Key informants were invited to participate by email and three attempts were made to reach each key informant. Key informants from eighteen hubs (75%) agreed to participate. In three cases key informants requested to bring additional team members to the interview, because they had specific knowledge of programmatic elements. As such, the total number of key informants was 24.

### Materials and procedures

The interview guide was developed by the study PI in collaboration with a team of investigators (LSM, RL, JAF, JH, CAB, RH) focused on community engagement, health equity, regulatory affairs, immigrant health research, and clinical trials engagement. The guide consists of seven semi-structured items exploring: (1) efforts to engage speakers of languages other than English as well as individuals with limited English proficiency, (2) organizational policies or procedures put in place to support efforts, (3) how efforts are monitored, (4) measurement of impact, (5) how organization culture influences efforts and is influenced by the efforts, (6) the relationship between community context and efforts, (7) lessons learned and recommendations for other hubs. Key probes explored specific engagement strategies, the impetus for implementation, and key implementation partners.

The PI and doctoral student conducted interviews with key informants via Zoom. At the onset of the interview, the purpose of the study was explained, and consent to record the interview was obtained. In cases where the consent to record was not obtained (*n* = 2), detailed notes were taken by the interviewer. The duration of the interviews was 20–40 minutes.

### Data management and analysis

Seventeen of the 18 CTSA hubs that agreed to participate completed the key informant interviews. Transcripts were generated for each interview using Zoom. CB listened to each interview and corrected inaccuracies in the transcription (in two of the 17 cases recordings were not made and researcher interview notes were analyzed). Documents were uploaded into NVivo 12 [17], and thematic analysis was performed [18]. LSM and CB read through all the transcripts multiple times. Four of the 17 transcripts were then used to identify themes in the data. Each of the two coders worked independently and then met to discuss identified themes, which were used to develop codes [18]. The codes and narrative descriptions were used to develop a codebook in NVivo that was applied to text segments across the 17 transcripts by two coders [18]. Discrepancies in the coding were identified by merging coded files and discussing instances in which agreement was below 80%. Seven parent codes and seven child nodes were identified, which can be seen in Table 1 Codes. When coding was complete, reports were generated for each code and summary paragraphs. Text segments from the data were selected to illustrate each of the identified themes.

**Table 1.** Codes

Codes
1. Approaches to language inclusion
1.1 Researcher driven.
1.2 CTSI services.
1.3 University services.
2. Strategies for language inclusion
2.1 Embedded Community liaison.
2.2 Navigators/Ambassadors.
2.3 CAB and partnerships.
2.4 Outreach.
3. Policies and programmatic changes to support the work.
4. Organizational impacts of the work.
5. Challenges
6. Lessons learned
7. Evaluation and monitoring

Strategies to increase linguistic diversity identified through the analysis were shared back with key informants. Fourteen key informants expressed interest in participating in the interpretation and dissemination of study findings. Based on the feedback provided by key informants, identified strategies that emerged in the data were organized using a framework developed by Warnecke *et al* (2008) [19]. We report on findings related to strategies employed by hubs to advance linguistic inclusion that were identified in the context of the framework. We then present challenges and lessons learned described by key informants.

### Findings: CTSA hub-level strategies to include participants with limited English proficiency

**Figure 1.**
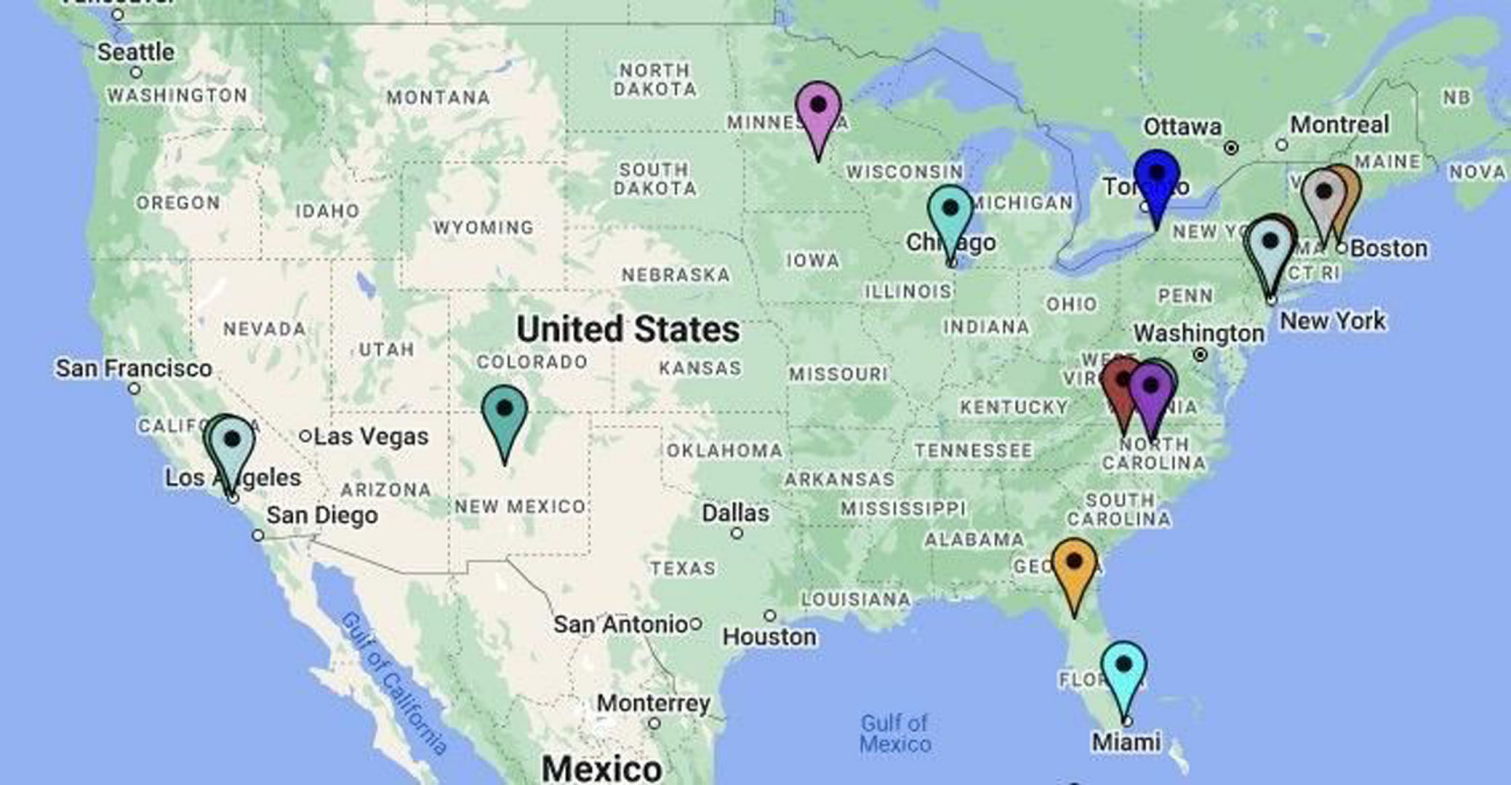
Map of Clinical Translational Science Award hubs.

We interviewed CTSA Hubs from across the country. The geographic distribution of CTSA hubs who participated in the assessment can be seen in Figure 1.

Most key informants were affiliated with the community engagement (CE) program or the special populations workgroup. Key informants had a variety of titles which included: core director, manager, investigator, or health educator; in one instance the key informant was a CE core director and MPI.

Key informants described several strategies intended to increase the linguistic inclusion in clinical and translational research at their hub. Overall, strategies described were driven at researcher, CTSA hub, and university or health center levels. Researcher-driven approaches relied on individual investigator or research group connections and resources (e.g., individual research programs). Those initiated at the hub level included services and initiatives specifically developed by the CTSA hubs to facilitate inclusion (e.g., translation, guidance, materials, staffing, and programs). Finally, there were university or health center-driven approaches designed to support inclusion (e.g., interpreter services and workforce development initiatives).

Hubs typically use multiple strategies to advance linguistic inclusion in clinical and translational research. Through interviews, we learned that strategies were interconnected, as illustrated by this quote:

*…we are trying to be as inclusive … as possible. …we do this in a few ways. I manage our recruitment and engagement center for the CTSI, …we meet with study teams to help them plan. So, looking at their protocol, … their inclusion and exclusion criteria, making sure that … their study isn’t excluding certain populations…. we work with them to really scrutinize that to see [if it] is really justified. …our regulatory team at CTSI has specialists that …can help site teams walk through the process and we collaborate with our … Human Research Protection program to help teams get connected with translation services …who are … able to certify the translation of the materials and consent form and then we work with our hospital partner for … interpreters, which are free through our partnership with [name of health system]. … If a study team is recruiting from the community broadly then they do not have access to the same type of connection through our hospital partnership, and so what we try to do is promote a diverse hiring of research staff. …That’s something we’re working more and more on, especially if the study … condition disproportionately affects a particular population [in our area].* (HUB13).

**Figure 2.**
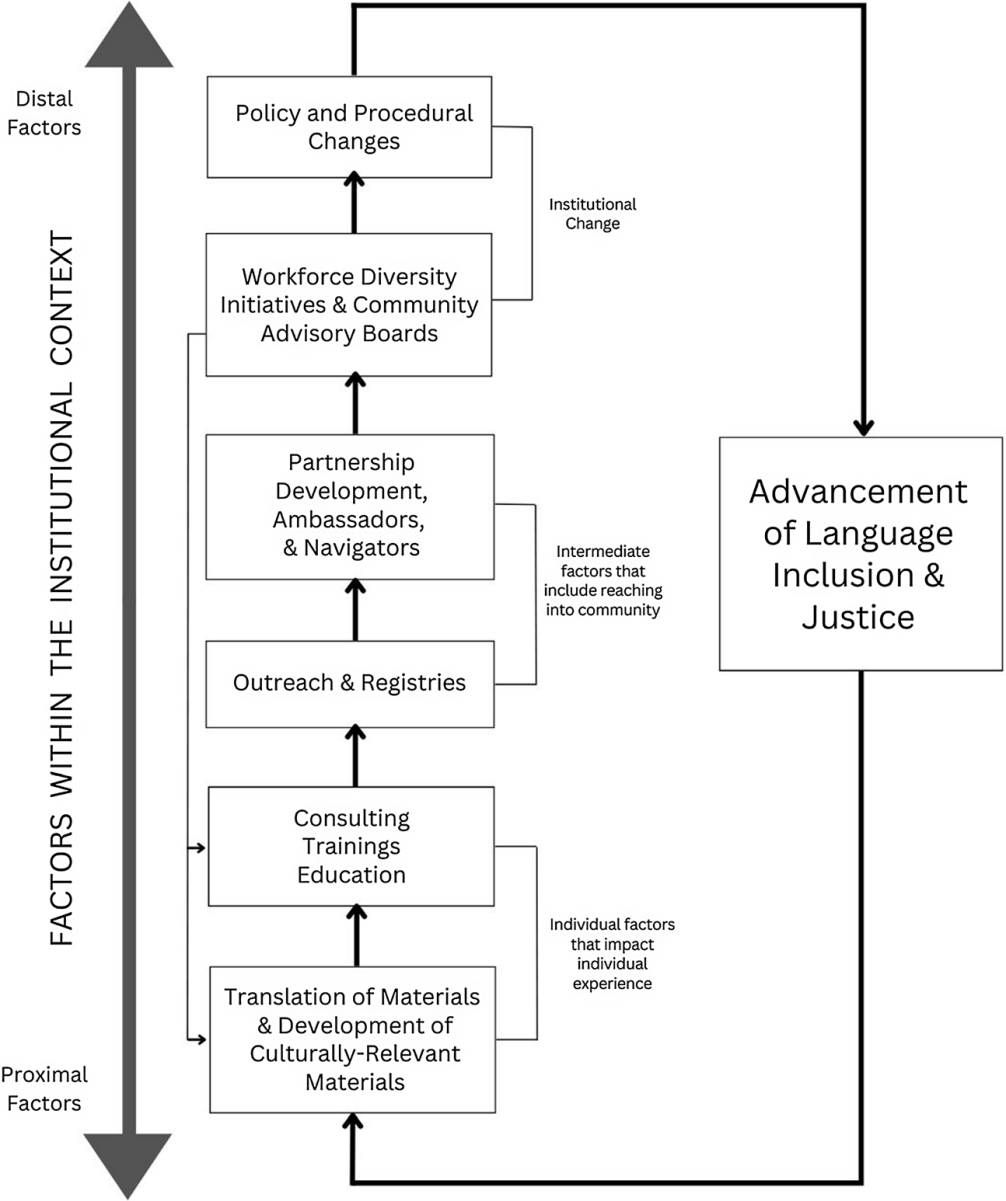
Strategies to advance language inclusion and justice in clinical and translational research.

We organized strategies that emerged in the thematic analysis based on Warnecke et al (2008) [19]. As seen in Figure 2, *Strategies to advance language inclusion and justice in clinical and translational research* there are proximal, intermediate, and distal factors for advancing linguistic inclusion being implemented at hubs we interviewed [19]. Proximal strategies are focused on service provision (translation and the cultural adaptation of materials) as well as consultation and services to improve researcher readiness for language inclusion. Intermediate strategies are focused on the external environment and designed to leverage community expertise through outreach, engagement, network building, navigation, and partnerships. Distal strategies at the macro level are focused on institutional change. These strategies included community advisory boards informing institutional activities as well as changing institutional policies and procedures related to workforce development, hiring and compensation, and institutional review.

### Consultation services for researchers

Key informants described providing consultation services to researchers to promote linguistic inclusion in several ways. Consultation services included reviewing and monitoring protocols to ensure representation of minoritized populations and the use of gender-affirming language, providing funds for translation, and making connections to community-based organizations to assist with language access as well as to ensure the cultural relevance of protocols. Some key informants reported their IRB website provides guidance to encourage researchers to assess the extent to which local communities are represented in their research, regardless of language spoken. In addition to one-on-one consultations, such education is also incorporated into training programs.

*…our community engagement core works closely with our recruitment and retention unit and our integrating special populations unit. And so we often get consultations from, … one of those groups, but we’ll work together to address them, and often one of the first issues is around, …[the] recruitment and engagement of diverse participants in trials, … one of the first considerations we all always provide consultation on who is the target community you’re looking to engage and, have you considered [the] cultural and linguistic context, … our preliminary consultation, guidance is around … accessibility of recruitment and outreach materials in terms of language, access, reliability, health literacy. … we’ve all also created some guidance documents around community engagement and that process of working with partners. … we provide those guidance documents to investigators.* (HUB 7)

Key informants also elevated the need to address social factors that can disproportionately impact populations they are working to include, and which intersect with language.

*I’m advocating for more funds to become available for that and for other issues that might be related to special populations as well. Transportation vouchers… for example, things …along those lines…*(HUB 8)

### Advisory boards

Many key informants referenced having permanent advisory boards. Advisory boards had different names and roles across sites but were all bodies designed to guide research engagement, partnerships, and inclusion. Many had linguistically diverse representatives. Others had advisory boards that advised researchers on translation resources and or reviewed translated protocols to ensure cultural relevancy and linguistic accuracy. One site key informant spoke specifically about examining documents to ensure cultural appropriateness using an intersectional lens.

*… both organizations serve on our Community Advisory Board. … We created the infographic collectively, and our CAB had lots of input …[on] what it said, and the wording….* (HUB 12).

Sites also reported contracting with advisory board agencies to provide translation services. Overall, advisory boards were seen by sites as an important strategy for leveraging community assets and leadership to advance equity and inclusion in research.

Some sites used the Community Engagement Studio model [20] as a strategy to help researchers plan. The Studio model is a one-time-only advisement session. For this reason, community advisors are intentionally selected to provide this service for specific research projects.

### Partnerships and outreach

Key informants frequently described conducting outreach and building partnerships in the community. This involved being present in the community and more importantly being part of the community. Multilingual CTSA staff were described as attending community meetings and events to familiarize themselves with local priorities and people. This work was not always associated with specific research projects but was intended to build bridges between the institution and the community to inform research priorities and practices.

CTSA hubs employed multiple outreach tactics including being present at community meetings and events. For example, one site described having a mobile unit that brings generalized services, resources, and health information to the community and provides an avenue through which researchers can connect with residents about both their priorities and research opportunities.

*…we have these mobile vehicles that we call Game Changers… because of this idea that we would go into communities and bring research, opportunity, and enhance, offer equity and research through that model. Well, that Game Changer is outfitted now with EPIC, which is our institution’s electronic health record. … it matters because one we can push research notifications to people who have MyChart. But what I was mentioning before is that our consent to contact and our broad consent initiatives … will be integrated into the registration for EPIC. So, people in communities who are interacting with these outreach vehicles are going to be asked. “Do you want to participate in consent to contact? Do you want to participate in broad consent?” [They will] have the opportunity to engage with community health workers …around why this matters, why diversity and science matters, and we are expanding enrollment … beyond those who come to our health system ….* (HUB 14).

Hub extensions – or additional CTSI locations set up within a community away from the university – were another outreach tactic. In these cases, multilingual education hub staff were embedded in areas where most residents spoke languages other than English. For example, we heard from hub 6 that they had an extension site two hours away from the University in an area predominantly populated by members of the Latinx community. Hub staff at the extension site serve as liaisons between the CTSI and the community, focusing on rapport building and relationship development. As liaisons, they also educate the CTSI about community priorities, educate community members about research opportunities, and provide health education.

Ambassadors, navigators, and research *promotores* were also used for outreach and research education as well as to facilitate engagement and partnership development in communities. These were individuals hired by CTSI hubs as cultural and linguistic brokers to champion research in the community as well as to help community members navigate the research process within the institution.

*…we actually built-in community outreach liaisons. And so, because what we found is that we were getting requests all the time from investigators, saying…, we need a Spanish speaking staff to help us with, to lead this focus group or to help us with recruitment or to do X.* (HUB 7).

*Something that’s worked for us is having community champions. You can call them patient advocates in some ways as well, but also with someone in the community that is really engaged with us, really understands what studies are coming up and engaging with the communities to help enroll and recruit for our studies at [our hub]…* (HUB 15).

At some hubs, they also train clinical research staff to better understand community and cultural context.

*…what we …have had our community health workers train our clinical research coordinators to understand the multi-level barriers that patients may bring to research encounters, some of which are, you know, maybe linguistic, maybe around access to the formal education system, may be [cultural], and so that the knowledge from being on the ground translates to how we approach research opportunity within clinical settings.* (HUB 14).

### Registries

CTSI hubs described the use of research registries to track and connect with populations that speak languages other than English as an important resource for advancing linguistic inclusion. Registries were built and maintained through outreach and often involved community liaisons and navigators as well as the research staff. Registries serve multiple purposes such as supporting recruitment and study engagement; informing community engagement and capacity-building efforts; and developing relationships as well as monitoring and evaluation. Registries were not only used for research recruitment; they were also used for information and resource sharing as well as educational efforts. Registries were dynamic, updated frequently, and constantly used to assess CTSI efforts and explore change over time. Registries allow sites to build relationships and assess how they are doing with gaining community trust.

*…. we are linking people [on the registry] to research studies. But of course, in our work it’s all about like the relationship building and breaking down that [mis]trust. … we measure trust, right now it’s on a scale of one to 10. How much do you trust researchers on the scale of one to 10. We collect that data when we first administer a health needs assessment and collect that baseline, we do a 60 day follow up, and 120 day follow up for that as well. Part of that is just any tailored referrals that we gave to them is wanting to know if there are barriers to using any of those referrals, what those were, if at all. If there are any other referrals that they may need, and then re-measuring those trust measures during those follow-up calls as well. So again, all of that is tracked [in the registry]* (HUB 16).

### Procedural and policy change

Sites shared organizational policy and procedure changes that were needed to facilitate inclusion. These changes were focused on the institutional review board (IRB), sponsored programs, human resources, and finance. With respect to the IRB, CTSI hubs describe the importance of having IRB guidance to prompt researchers to focus on linguistic inclusion by providing resources as well as listing frequently asked questions and guidance on forms and applications. They discussed the importance of having IRB training available in multiple languages to support a multilingual research team.

*I think it says something because you have me [in a leadership role] as somebody who’s more community minded. [I have been able to advance a number of initiatives]… for human subjects research training we worked with [another] CTSI [that] …developed a program called CIRTIfication* [21] *and we adapted it in Haitian Creole… it’s all of those kinds of things that we try to do.* (HUB 14).

In relation to sponsored programs and grants management side, CTSI hubs thought it was important to educate teams on how to develop grant budgets in a way that facilitated the hiring of multilingual project staff and considered the cost of translation. Some CTSI hubs provide this education through consultations but felt guidance would be more effective if provided at the institution or systems level. Others made guidance documents and resources available on the grants administration page.

Human resources (HR) were discussed by most CTSI hubs as an important interest group. Although most sites were not working directly with HR, they noted it as an important next step in their work. Sites that were working with HR advocated for changes to job postings as well as for the intentional distribution of said materials to ensure they reached diverse audiences. Hubs also elevated workforce development efforts to hire and train shared pools of research staff as well as efforts to develop career ladders. In some cases, this included a focus on the career transition from ambassador to research coordinator to project manager. Hubs described challenges associated with both retaining and promoting multilingual research staff.

*My other hat is in the workforce development and training portion of CTSI. …we do see all new coordinators through our onboarding program and encourage, … hiring a diverse workforce. We’re planning some conversations, discussions with managers on how to do that. …We meet with managers on a monthly basis from across the university. So, all the research managers, or maybe about 30, to really talk about many things that come up. But hiring is certainly something that comes up often and what are some of the best practices looking at and how the jobs are posted. …. It’s really the hiring manager who works with HR. … we can provide education. We can provide materials, but we do not have ultimate say over who gets hired.* (HUB 13)

*…we’re giving the weight to the ability to communicate in multiple languages. As you know, [it is a] meaningful skill that… needs to be compensated. …. But something that … I do not think … automatically translates into the pay scales, and … the existing job descriptions.* (HUB 10).

Sites consider financial arrangements and partner compensation policies to be critical to advancing the work of linguistic inclusion in research. Although community partners are critical to the success of engagement and recruitment efforts, compensation can be a pain point. Sites discussed the importance of partner compensation policies. This involved working with administration to revisit contracting policies that are more applicable to large institutions than grassroots organizations.

*…I …sometimes leverage my muscle as the Vice Provost to address commonly articulated constraints by community members participating in research. So, as an example, let’s say you want to subcontract with an organization, and they’re going to hire… staff, that will be instantly involved in your research implementation. Well, we cannot ask those small community-based organizations to carry the same kind of insurance coverage that we might ask … a multinational corporation to provide… We cannot assess risk from the same framework and we cannot be delayed in providing payment, because it affects their ability to pay staff, and oftentimes their budgets are not robust enough to pay staff … doing work outside of … their immediate mission. So that’s something easy that we’ve done right.* (HUB 14).

### Challenges and lessons

The main challenges to linguistic inclusion sites described encountering arose from cultural procedures in research that normalize the exclusion based on language proficiency, researchers’ disengagement with communities, lack of financial preparation, and culturally appropriate language translations. Key informants also cited a linguistically diverse, more representative workforce as necessary to ensure linguistic inclusion.

Key informants pushed back against the idea that not speaking English is reason enough for exclusion from research participation. Multiple institutions mentioned efforts to question these assumptions but also expressed frustration with the process.

*And I do believe strongly that non-English speakers are an example of that, you know so whenever we do a consult, and we see somebody put non-English as an exclusion criterion. We always have to ask the question of why. And usually the answer isn’t sufficient, and we start to consult with them about it. We really should anticipate the language barriers that you might anticipate and get out ahead of that by doing some translations. One challenge we do run into is studies that use validated instruments that have never been translated. You can’t just start translating surveys that have been previously validated. So that becomes a barrier sometimes, and that’s an area of interest for us to think more about how to do that.* (HUB 5).

With that in mind, one site described partnering with their institution’s IRB to explore patterns related to exclusion.

*…in our center we’re trying to affect organizational culture when and as I mentioned to you, with the way that we’re trying to do things in terms of how we communicate, presenting everything, at least in 2 or 3 languages, doing the outreach in 2 or 3 languages, and with our study to work with the IRB to start changing language, inclusion, practices like from our little center. No one’s going to listen to us. but he would get the IRB to say, oh, we need to pay more attention when you send your investigator study plans. You need to explain ad nauseum why you are excluding them based on language. Then I feel like that’s going to put potentially initially a pressure that people might not like. But sometimes cultural change involves people. Yeah, yeah, changing cultural practice. People will get upset. That’s correct. But that’s why we want to make an effort to document these so that there’s not an argument that we’re just coming up with, not, because we’re nice, you know. We want to systematically make sure that as research teams we use evidence to support some of those changes.* (HUB 10)

Key informants discussed issues of culturally appropriate translations and financial resources as vital for linguistic inclusion. Key informants across sites reported cultural tailoring and translations needed to be in the budget (the organization and researcher’s budget) in advance.

*… we can do professional translation. That means we have to put it into the grant right the cost of that but the cultural tailoring pieces also. So, it’s not just like maybe straight translation, but we have to do tailoring of content to patient populations, and when it goes beyond Spanish, I think it’s a little bit harder to find, to do the translations, you know. So, if it’s for a smaller group of patients …The resources are needed, I guess.* (HUB 3).

*… it’s really important to recognize that this actually requires investment of resources. And so, like translation is expensive, like, you know, like this is important.* (HUB 2)

Key informants described their thinking about culturally appropriate translations and budget issues.

*We’re actually putting together our resubmission, you know, for our renewal or our first mission for our renewal. And we’re really going to kind of try to enlist the aid of Hispanic organizations like we have a Hispanic League in. And so, you know, just bringing these individuals and these groups into advisory boards, you know, just to not just provide, you know, like language, translation services. But you know there might be, you know, particular needs of this Hispanic community around. Let’s say, a patient or participant navigation. You know things of that nature. So, you know, we’re planning to maybe include that into the renewal proposal. But we’re doing some things that they’re popular. You know. Most of our language requests are for Spanish. But you know, in some of our communities in the triad area.* (HUB 8).

Key informants struggled with research teams trying to recruit and enroll non-English-speaking populations without planning or creating relationships with the community first. These teams faced challenges. Key informants encouraged the development of early and meaningful connections with community partners prior to soliciting funding.

*One of the things that we really encourage them to do is that they shouldn’t just go to community organizations at the time of recruitment, but they should engage early enough in the process. so that you build a relationship. and that you can explore some of these needs are their language needs and their culturally based needs*. (HUB 10).

While many key informants reported that universities were making efforts to increase diversity to ensure the workforce was more representative of the community at all levels (i.e., faculty, research assistants, etc.), it was noted that work still needs to be done. Even in ethnically diverse parts of the country, their challenges were described.

*I think, where we struggle is ensuring that that diversity ends up moving to leaders … to levels of leadership, if you will, that ultimately have the potential to meaningfully affect change.* (HUB 14).

Additionally, institutional barriers to community participation in research include issues of trust (or distrust and mistrust).

*I would add just one more thing to it, just like the historical context as well. I think that’s real because we work for [University X]. And there might have been history, or past trauma, over past 60 variances and other communities that have had those connections with [University X]. So, even though it was not us who did, even though it was like generations ago. There’s still that memory there, and so to be knowledgeable, and being able to respect that, and not just feel like, “Well, I”m not that person,’ you know. (…) I think us, acknowledging that in us having those past experiences, and we could bring that to the table again. It just builds on that trust and acknowledgment and respect, and just being able to meet people where they are and what they’ve gone through. I didn’t want to forget that.* (HUB 4).

Finally, key informants described unnecessary bureaucratic processes as a barrier to hiring a more linguistically diverse workforce.

*…HR, for example, a community member who has a past history of incarceration. I’m, trying to push HR, I just had a whole issue about hiring a community health worker and [one had] a history of incarceration, and I was told that … my best candidate was ineligible for hire, even though their crime did not pose risk to the health and safety of others. They [HR] want certain qualifications that [can exclude top candidates] … Qualifications I’m looking for…---Are you a natural helper? Do you have a large social network? Do you speak languages [prevalent in the community]? Are you able to mobilize people? [Do] you know our community partners?* (HUB 14).

*Yeah, you know, I mean, there’s so many. You know, whole other conversations and something we’re working on is educating the IRB about community engaged research because it’s not designed to evaluate that it’s designed to evaluate clinical trials. You know, and there’s just so many barriers to engagement of external partners in the research process … Yeah, some of the metrics that the IRB is assessing, or should be more about like research team composition. And you know how well you are able to match the needs of your participants with your study.* (HUB 7).

### Evaluation and monitoring

Key informants described very different approaches to evaluation and monitoring. Most tracked the provision of consultation services. Some relied on analytics for views and downloads of resources, and a few had dashboards with research participant outreach counts; however, these counts could not be filtered by language. Key informants agreed more work needs to be done to evaluate and monitor efforts to advance linguistic inclusion.

*… we monitor is really by tracking consultation, you know, we track consultations and outcomes associated with consultations we are now tracking, … This is kind of like how we work together. We’re tracking … if we provide a consultation, We’re tracking kinds of recruitment progress over time. And now we’re looking at diversity of participants with some of those changes we’re making that just started this year.* (HUB 7).

*…I’m trying to get some data added to our CTMS, our clinical trials management system, which we use on core about whether what studies are available and which languages we are tracking on dashboards who are participating in terms of some demographics like race and ethnicity and age and gender. But we are not tracking which language in a way that I can track on it. Study teams are, but it’s not. I can’t see it aggregately yet. so, I’m trying to see what kind of fields could be added to understand which studies are available beyond English in a non-short form version, right? So, the short form can be used. But what studies are actually planning proactively have their documents translated, and have you know, the process for enrolling non-English speaking participants or in certain languages, and then can we display those on something like our tool called Study Finder.* (HUB 13).

## Discussion

Through key informant interviews, we found hubs were engaging actively in efforts to advance linguistic inclusion in clinical and translational research. Most efforts were focused on Spanish speakers; however, hubs recognized the need for broad linguistic inclusion. Hubs described barriers consistent with the literature such as trust, language, attitudes, beliefs [22,23], the availability of language-concordant materials, cultural and linguistic competence of staff, and limited outreach to a shared understanding of the benefits of clinical trial participation [24,25]. They also identified a number of strategies to overcome these barriers which included building researcher skills and systems change efforts, which are also described in the literature [26].

Systems-level strategies were focused on hiring and workforce development, which have also been discussed in the literature [14,27]. Hubs acknowledged that although efforts are underway, achieving language justice and inclusion will require major system-level changes. They discussed barriers associated with building a workforce reflective of the linguistically diverse populations in their catchment areas. These challenges included hiring, retention, and advancement. Consistent with the literature on community health workers (CHW), hubs reported wanting a workforce with the multicultural competence skills to engage the community members [28]. HR requirements posed a barrier to this goal. An implementation science evaluation of a CHW intervention described similar HR requirements related to licensing and education as a barrier to CHW recruitment as well as hiring; meanwhile, post hiring institutional policies related to liability were found to limit the engagement in the community [29]. Nonetheless, there are important lessons to be learned from the CHW literature as it relates to creating career ladders and the integration of a diverse workforce (CHW) [30,31].

This exploratory study is not without limitations. We report on efforts at only 17 hubs. It may be that there are additional hubs doing work to advance language access, inclusion, and/or justice that we did not identify, given we scanned hubs funded in 2020 and 2021. It may also be that language inclusion efforts are not highlighted on hub websites. Moreover, only CTSA hubs were included. Understanding what non-CTSA institutions are doing may broaden the scope of best practices. Additionally, we interviewed key informants with specific knowledge-related efforts focused on linguistic inclusion. In some cases, multiple informants participated in the interview to cover specific aspects of programing. It may be that additional efforts unknown to the key informant were missed. Finally, we did not collect demographic data from key informants. As such, it may be that there are unknown demographic factors that influenced our findings. Despite these limitations, multiple efforts focused on linguistic inclusion were identified. In addition, through this process, we have built a network of champions with an interest in advancing linguistic inclusion.

### Recommendations

The change work needed to advance linguistic inclusion will not happen overnight. It is likely to be both difficult and costly and must recognize that iterative and intentional efforts will be required to sustain any demonstrated improvements. The CTSA consortium allows sharing of best practices and a concentrated focus on the topic. This intention may facilitate the rate of change. Based on these lessons learned at 17 hubs, we provide the following recommendations generated by coauthors from 15 hubs:

1. Share best practices, guides, and materials. CTSA hubs can circulate best practices among themselves and within their institutions. Post best practices on IRB and sponsored programs research pages and embed them in training programs as well as new faculty and investigator orientation materials and consultation services. This will address the challenge of investigators and teams not having information about how to budget and prepare for linguistic inclusion.
2. Allocate funds for language inclusion. CTSA leadership and investigators can encourage federal funders, industry, and regulatory bodies to ensure funded grant proposals include budget line items specific to language access by including requirements in request for proposals related to inclusion. CTSA leaders and funders alike are in a unique position to shape behavioral norms among investigators through their guidance and expectations.
3. Advance equitable hiring practices. Key informant elevated the importance of hiring equity in advancing linguistic inclusion. Ways to ensure institutions are reflective of the local populations include assessing the local landscape, monitoring demographic shifts, and building bridges in communities to ensure pipelines are in place to cultivate a representative workforce at all levels and building internal capacity for diversity equity, and inclusion to increase retention.
4. Invest in advisory boards. Convene and compensate community advisory boards to provide consultation to CTSA and institutional leadership on policies, procedures, and activities. Key informants spoke to the importance of connecting to communities early in the research. Advisory boards are one way to maintain an ongoing relationship with communities to engage communities in meaningful governance.
5. Assess and continuously monitor researcher readiness for ethical engagement with linguistically diverse communities using training formats like those used for conflict of interest and human subjects’ trainings.

## Conclusion

Language justice is paramount to *bringing more treatments to all people more quickly.* Inclusion will require institutional transformation and CTSA hubs are well positioned to catalyze change. Some are already implementing strategies to advance inclusion. There is a need for rigorous evaluation of their efforts. In addition, CTSA hubs cannot do this work alone. There is an important role for federal funders, industry, and regulatory bodies to hold institutions accountable.
